# The effects of strain history on aortic valve interstitial cell activation in a 3D hydrogel environment

**DOI:** 10.1063/5.0138030

**Published:** 2023-04-03

**Authors:** Toni M. West, Daniel P. Howsmon, Miles W. Massidda, Helen N. Vo, Athena A. Janobas, Aaron B. Baker, Michael S. Sacks

**Affiliations:** 1 James T. Willerson Center for Cardiovascular Modelling and Simulation, Oden Institute for Computational Engineering and Sciences and the Department of Biomedical Engineering, Austin, Texas 78711, USA; 2 Department of Biomedical Engineering, The University of Texas at Austin, Austin, Texas 78711, USA

## Abstract

Aortic valves (AVs) undergo unique stretch histories that include high rates and magnitudes. While major differences in deformation patterns have been observed between normal and congenitally defective bicuspid aortic valves (BAVs), the relation to underlying mechanisms of rapid disease onset in BAV patients remains unknown. To evaluate how the variations in stretch history affect AV interstitial cell (AVIC) activation, high-throughput methods were developed to impart varied cyclical biaxial stretch histories into 3D poly(ethylene) glycol hydrogels seeded with AVICs for 48 h. Specifically, a physiologically mimicking stretch history was compared to two stretch histories with varied peak stretch and stretch rate. Post-conditioned AVICs were imaged for nuclear shape, alpha smooth muscle actin (αSMA) and vimentin (VMN) polymerization, and small mothers against decapentaplegic homologs 2 and 3 (SMAD 2/3) nuclear activity. The results indicated that bulk gel deformations were accurately transduced to the AVICs. Lower peak stretches lead to increased αSMA polymerization. In contrast, VMN polymerization was a function of stretch rate, with SMAD 2/3 nuclear localization and nuclear shape also trending toward stretch rate dependency. Lower than physiological levels of stretch rate led to higher SMAD 2/3 activity, higher VMN polymerization around the nucleus, and lower nuclear elongation. αSMA polymerization did not correlate with VMN polymerization, SMAD 2/3 activity, nor nuclear shape. These results suggest that a negative feedback loop may form between SMAD 2/3, VMN, and nuclear shape to maintain AVIC homeostatic nuclear deformations, which is dependent on stretch rate. These novel results suggest that AVIC mechanobiological responses are sensitive to stretch history and provide insight into the mechanisms of AV disease.

## INTRODUCTION

**FIG. 1.**
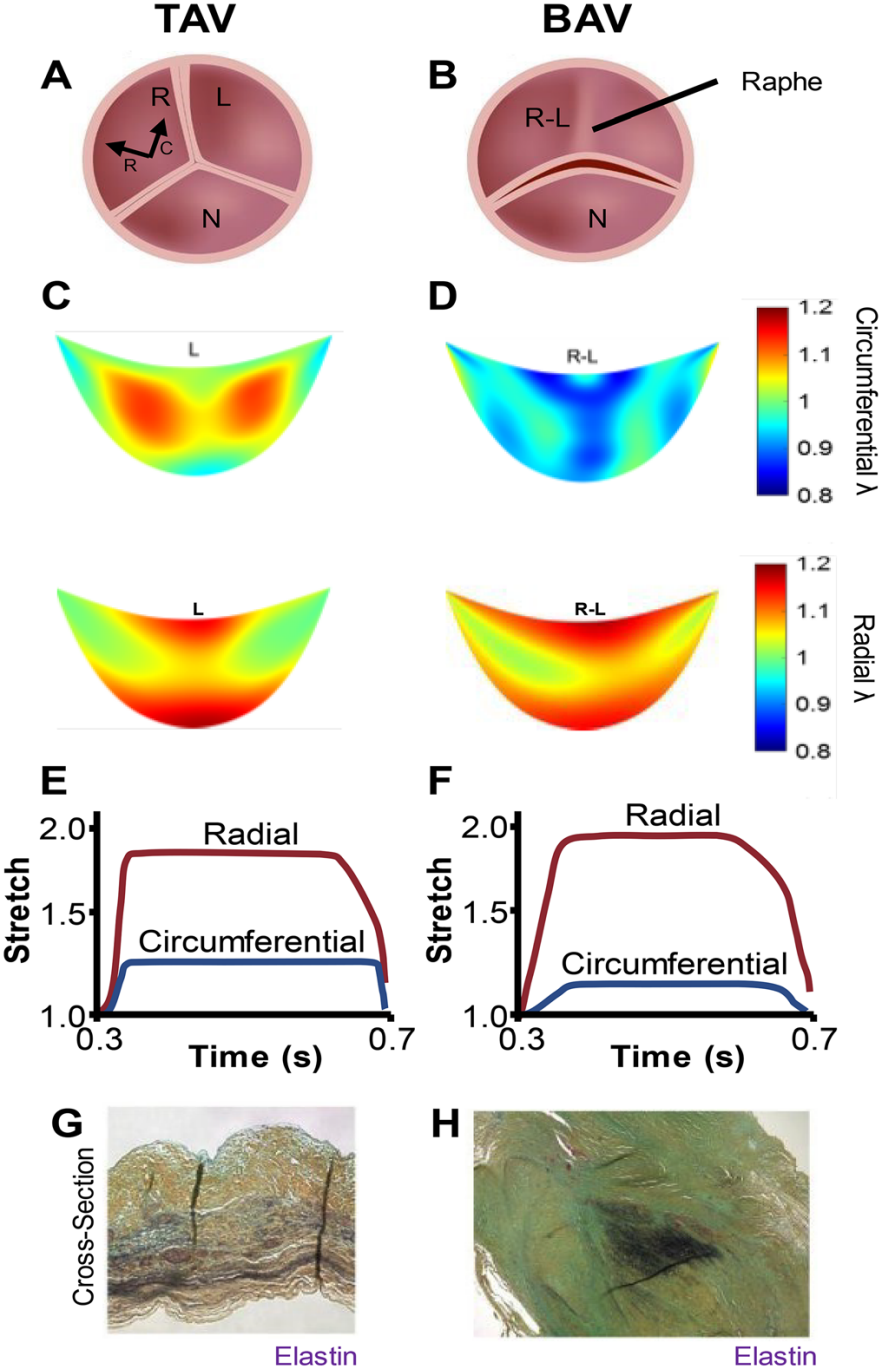
Multiscale nature of TAV and BAV function. (a) and (b) Renderings of a (a) TAV and (b) BAV during diastole. The Raphe is the area where fusion of two leaflets has occurred in a BAV. Circumferential (C) and radial (R) directions are depicted. (c) and (d) Mean circumferential stretch fields and radial stretch fields for (c) left (L) TAV leaflets and (d) right–left (R–L) fused BAV leaflets, as calculated from human *in vivo* echocardiogram measurements in Ref. 2, depict that the peak circumferential stretch field is largely reduced and the peak radial stretch field is slightly increased in BAVs. (e) and (f) Overview of data from time vs average stretch plots of (e) porcine TAVs in *ex vivo* flow loops,^1^ and (f) computationally simulated BAVs^5^ derived from data collected in Ref. 1 indicate that in BAVs, there is a large reduction in peak circumferential stretch and root mean squared value of the waveform, slight increase in radial stretch and root mean squared value of the waveform, and a decrease in both circumferential and radial rate of stretch. (g) and (h) Histology of cross sections of bicuspid aortic valve leaflets depicting that (g) highly ordered layered ECM patterning in the body of the noncoronary (N) leaflet and (H) disordered ECM clumps in the Raphe (modified from Ref. 33) correlate with changes in stretch waveform.

Upon each heartbeat, the aortic valve (AV) undergoes large, highly dynamic cyclic deformations, with a total lifetime number often above 3 × 10^9^ cycles. Normal functioning AVs are formed from three distinct leaflets (hence the term tricuspid AV or TAV), [Fig. 1(a)]. During diastole, the TAV closes rapidly in approximately 50 ms, remains closed for about 600 ms, and then reopens just as fast in approximately 50 ms.^1^ During the relatively long duty cycle of stretch that occurs while the valve is closed, leaflet tissue stretch is anisotropic, with a circumferential stretch of λ_c_∈[1.00–1.15] and a radial stretch of λ_r_∈[1.00–1.20], depending on the region [Figs. 1(c) and 1(e)].^1,2^ The embedded aortic valve interstitial cells (AVICs) within the valve leaflets are consequently subjected to large deformations over the cardiac cycle.^3^ Of many forms of AV diseases, those involving the bicuspid AV (BAV), where two leaflets form instead of the usual three,^4^ are of particular importance. Although a spectrum of BAV anatomies occur, the most common configuration occurs when the left (L) and right (R) leaflets are fused to form one large R–L leaflet that interacts with the morphologically normal noncoronary (N) leaflet [Fig. 1(b)].^4^ We and others have recently quantified that there are major intra- and inter-leaflet differences in deformation that occur between BAVs and TAVs, which include differences in both stretch levels and stretch rates.^1,2,5^ Approximately 50% of aortic valve replacements occur in BAV patients, even though BAVs only occur in 1.4% of the population.^4^ Although aortic stenosis (AS) and/or calcific aortic valve disease (CAVD) develop in older adults with normal TAVs, BAV patients typically progress more quickly into disease that requires valve replacement decades earlier.^6^ Current treatments for AV disease remain almost exclusively AV replacement with a prosthetic device, although AV repair has sometimes been utilized.^6^ Early development of AV disease, coupled with the limited durability of AV replacements, require BAV patients to undergo multiple prosthetic replacements in their lifetime, creating a very high procedural burden and increased mortality risk.^7^ While no pharmaceutical therapies for treating valve diseases exist, such therapies would alleviate the high procedural burden in individuals with BAV. AS and CAVD are both characterized by stiffening and thickening of the leaflet, causing improper leaflet coaptation and regurgitation. As these diseases progress, it is possible that a vicious cycle occurs where the resultant regurgitation may decrease stretch imparted on the valve, possibly activating the resident fibroblast-like AV interstitial cells (AVICs) that maintain the valve to make the valve even stiffer. Thus, improving understanding of how changes in 3D mechanical effectors in the valve relate to cellular biochemical signaling can help to identify new targets for drug development for AS and CAVD.

Unlike the cellular basis of disease in other components of the cardiovascular system, less is known about how mechanical signals are transduced to AVICs in health and disease. Activation of AVICs into myofibroblasts is a telltale sign of AV disease from mouse to human, with activated AVICs producing large amounts of ECM, matrix remodeling enzymes, and cytoskeletal components.^8^ AVIC activation is associated with increases in cytoskeletal rearrangement including alpha smooth muscle actin (αSMA) and vimentin (VMN) polymerization. Buildup of αSMA-positive F-actin filaments increases VIC stiffness and contractility.^9–13^ Heightened contraction in activated AVICs is detectable at the tissue level and is often associated with ECM pathological remodeling.^13–15^ Interestingly, VICs isolated from heart valves from the left heart, where tissue stress levels are higher, are stiffer, contain more αSMA, and have more contractile potential than VICs from the right heart valves.^16,17^ VMN is also more highly expressed in valves of the left heart than in those of the right heart^18^ although it is only expressed at low levels in healthy adult VICs.^19^ VMN is increased in valve interstitial cells from leaflet explants when stretch and pressure exerted on the leaflet is varied to non-physiological values.^20^ It has also been observed that in fibroblasts and many other cell types VMN polymerizes to form a cage around the outside of the nuclear membrane to protect the nucleus from rupture when the cell is under large 3D stretches.^21^ Important nuclear transcription activators of VIC cytoskeletal and ECM components are small mothers against decapentaplegic homologs 2 and 3 (SMAD 2/3). It has been reported in mesenchymal stem cells and osteoblast-like MC3T3-E1 cells that SMAD 2/3 can be differentially activated by mechanical stretch without the exogenous addition of its canonical activator, TGFβ.^22,23^ Furthermore, cyclic stretch leads to AVIC-mediated collagen remodeling and mitral (M)VIC-mediated increases in VMN and ECM proteins.^24–28^ The above studies suggest that AVIC activation is sensitive to the specific patterns of *in vivo* mechanical deformation, yet there remains a substantial disconnect between the understanding of organ-level and cell-level deformations within AVs.

We recently performed studies on MV leaflets to explore the interconnection between tissue deformation and MVIC phenotype.^29,30^ In these studies, MV deformations that emulated surgical repair were transduced to the MVIC nuclear morphology, and this resulted in decreases in MVIC αSMA expression and collagen mass fraction, indicating that physiological deformations of the ECM get transduced to VICs, causing the VICs to adjust their expression of key disease state markers.^29,30^ In another work on AV, it has been reported that AVICs grown in elongated molds depict elongated nuclei that correlate with increased actin organization and endothelin-induced contractility.^31^ In addition, pro-calcific AVICs in CAVD patient leaflets near calcific nodules have atypical nuclear morphology when compared to healthy controls.^32^ Hence, VIC nuclear shape changes play an important role in VIC mechanobiological responses. The present study aimed to determine how specific properties of deformation affect AVIC morphology and phenotype over multiple cardiac cycles.

When the BAV closes, the area of leaflet fusion (the Raphe) often folds to produce a faux coaptation, causing the normal cyclic deformation patterns to be significantly altered. Specifically, measurements from human *in vivo* echocardiograms from BAV and TAV patients depict that the peak diastolic circumferential stretch field is greatly reduced in BAVs, with the most pronounced decreases occurring in the Raphe regions, while peak radial stretch fields are slightly increased in BAVs [Figs. 1(c) and 1(d)].^2^ Similarly, *ex vivo* flow-loop measurements of stretch in porcine TAVs,^1^ when modeled for differences in flow patterns in both BAVs and TAVs,^1,5^ show that peak diastolic values in the radial direction are slightly increased, rate of stretch is slightly reduced, and peak stretches in the circumferential direction are greatly reduced [Figs. 1(e) and 1(f)]. Moreover, since the peak stretches are greatly changed, the root mean squared (RMS) values of the circumferential and radial waveforms are also significantly affected, leading to the average overall radial and circumferential stretches that a valve experiences over a lifetime being greatly different between BAVs and TAVs. Upon disease progression, there is a marked change in collagen and elastin patterning and thickening within the ECM, which is coupled to a general thickening of all the valve leaflets especially near the free edges [Figs. 1(g) and 1(h)].^33,34^ The areas where there is ECM thickening and disorganization, which are most prominent in the Raphe of BAVs, correlate with areas in BAVs where peak circumferential stretch and RMS values of circumferential stretch are reduced most.^2,33^ Thus, altered stretch patterns in diseased heart valves correlate with AV disease progression. Yet, understanding which of the mechanical effectors of stretch leads to early disease onset remains limited, largely because studying the link between AVIC phenotype and deformation in native valve tissues is complicated by the optical opacity of valve tissue,^35^ making it nearly impossible to quantitate AVIC activation *in situ*. Similarly, studies of isolated AVICs on 2D substrates are complicated by AVICs behaving quite differently in 2D cultures than in their native 3D state. Specifically, gene expression profiles for AVICs grown in 2D are highly activated when compared to freshly isolated or 3D cultured AVICs,^36^ and contractile forces determined from AVICs cultured in 2D are more pronounced than those observed in 3D cultures.^11,37^

Based on the above considerations, the present work focused on determining how the mechanical cues of peak stretch, stretch rate, and average stretch value affected biochemical and cellular structural markers in AVICs. The use of 3D cultures allowed the deformations to be transduced to the imbedded AVICs, better emulating *in vivo* conditions. This was done using poly(ethylene) glycol (PEG) gel, which is an accepted simulacrum for soft tissues as it provides a 3D scaffold for cells that mimic many of the biochemical and mechanical cues known to exist in native ECM.^12,38^ PEG gels also reduce many of the confounding variables that are present in explanted tissues. For example, the gel stiffness and the number of integrin binding sites (i.e., RGDS) can be held constant independently.^11,39^ In addition, PEG gels are optically clear, allowing for high-definition microscopy of imbedded cells by immunocytochemistry (ICC) in a 3D environment. In the present study, a high-throughput AVIC-imbedded PEG gel experimental model system was utilized to investigate, for the first time, the effects of stretch rates and levels on AVIC activation state. Specific stretch waveforms were employed on these 3D PEG gel cultures to determine if peak stretch, stretch rate, or root mean square (RMS) stretch levels were important effectors of AVIC activation, as measured by αSMA and VMN polymerization and SMAD 2/3 activity.

## METHODS

### Experimental design overview

**FIG. 2.**
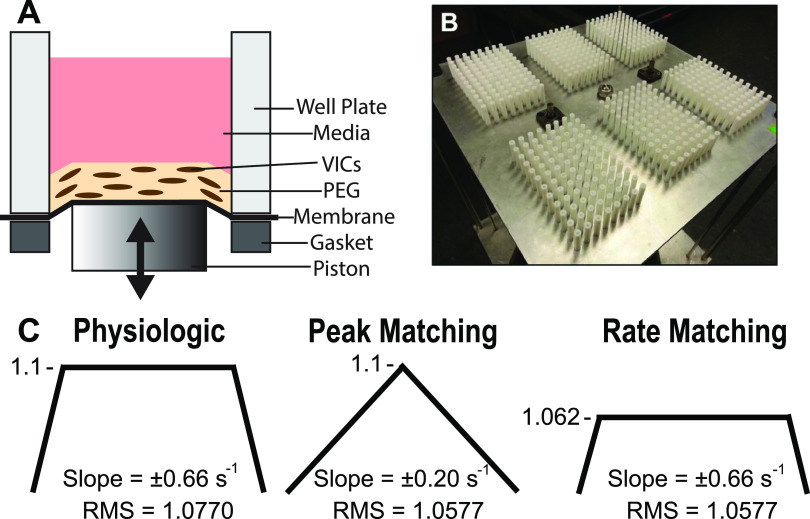
Experimental setup on the 3D high-throughput biaxial oscillatory stretch system (3D HT-BOSS). (a) and (b) The HT-BOSS was implemented to apply oscillatory equi-biaxial stretch up to six 96-well plates simultaneously by utilizing a linear motor that pushes pistons into the bottom of a specialized well plate made with a flexible silicone membrane bottom, which was functionalized to hold PEG gels with AVICs imbedded in them. (c) The 1 Hz waveforms applied by the HT-BOSS, with the physiologic wave moving to the peak stretch of 1.1 as quickly as the motor could do so, the peak matching wave moving to 1.1 within 0.5 s, and the rate matching waveform maintaining the same slope as the physiologic wave and the same RMS value of the peak matching wave, making the peak stretch 1.062.

The goal of this study was to investigate how specific stretch histories affect activation state of AVICs in a 3D environment. Porcine AVICs were imbedded in PEG gels and conditioned for 48 h on the high-throughput equi-biaxial oscillatory stretch system [HT-BOSS; Figs. 2(a) and 2(b)]. Conditioning waveforms were designed such that the effects on AVIC activation from stretch rate, peak stretch, and the root mean square (RMS) value of stretch could be distinguished. Furthermore, since changes in stretch seen in BAVs are more prevalent in the circumferential direction,^1,2^ values for peak stretch adopted for experimentation were based on those seen in the circumferential direction of AVs. Since normal TAVs experience square wave-like deformation patterns (Fig. 1),^1,2^ a similar waveform was implemented and termed the “physiologic” waveform [Fig. 2(c)]. The physiologic waveform achieved a peak stretch of 1.1 (i.e., 10% strain) in 0.15 s, resulting in a stretch rate of ±0.66 s^−1^ and an RMS stretch level of 1.0770. The RMS was calculated by taking the square root of the arithmetic mean of the squares of all points along the waveform. The “peak matching” waveform retained the peak stretch of 1.1, but with a stretch rate of ±0.20 s^−1^, and an RMS stretch level of 1.0577 [Fig. 2(c)]. The third waveform, termed “rate matching,” had the same shape of the physiologic waveform and the same stretch rate of ±0.66 s^−1^, but with the peak stretch value reduced to 1.062 so that the RMS stretch level would equal that of the peak matching waveform, resulting in the rate matching waveform having a time to peak stretch of 0.10 s [Fig. 2(c)]. By implementing these specific conditioning regimens, differences in activation due to stretch rates, peak stretch, and RMS stretch level could be dissected. Moreover, values implemented in the waveforms were chosen under the assumption that stretch history modulates AVIC activation over many thousands of cycles, and not because of single cycle events. This is analogous to findings from growth and remodeling studies, which are hypothesized to be VIC modulated.^40^ Methods were developed and verified for applying uniform biaxial stretch to 3D PEG hydrogel cultures and the AVICs imbedded within them to implement the above outlined experimental design in 3D cultures on the HT-BOSS.

### Cell sourcing and culture

Porcine AVICs were isolated, cultured, and expanded from physiologically normal wild-type hearts collected from a local abattoir as previously described,^41^ with the following modifications. Excised AV leaflets were soaked in phosphate-buffered saline (PBS) containing 1% penicillin/streptomycin for 10 min. The leaflets were then placed at 37 °C in 60 U/ml type II collagenase in PBS for 3 h and vortexed for 15 s. The resulting solution was removed to separate the endothelial cells from the remaining leaflet tissue. The remaining tissue was digested with 0.75 mg/ml collagenase in Earle's balanced salt solution for 60 min, vortexed for 1 min, filtered with a 100 *μ*m cell strainer, and centrifuged. AVICs were cultured and expanded in DMEM (Gibco) with 10% FBS (Gibco) and 1× penicillin/streptomycin/glutamine (PSG) on rat tail collagen-I (Gibco) coated plates. Passage numbers 4–6 were utilized for experimentation. All reagents and materials were obtained from Thermo-Fisher Scientific unless otherwise specified. The Institutional Biosafety Committee of the University of Texas, Austin, approved the implemented protocols (IBC-2021-00047) in accordance with NIH guidelines.

### 3D AVIC-hydrogel implementation on the high-throughput biaxial oscillatory stretch system (HT-BOSS)

Since AV leaflets and the imbedded AVICs undergo cyclical isotropic biaxial stretch, a custom-made device that we previously developed for stretching 2D cultures^42–45^ was further developed so that AVICs imbedded in 3D cultures could be mechanically conditioned. The HT-BOSS moves pistons against a flexible culture surface of silicone, causing the silicone membrane to be stretched equi-biaxially [Fig. 2(a)]. Briefly, the HT-BOSS employs a true linear motor with a resolution of 10 *μ*m that pushes a platen that holds the pistons onto the bottoms of 96-well plates. As many as six 96-well plates (or 576 samples) can be conditioned simultaneously with the HT-BOSS. The 96-well plates were produced by sandwiching 0.005″ flexible silicone sheets (Specialty Manufacturing, Inc.) between custom-made red silicone gaskets and aluminum top and bottom plates, which were held together by screws. Plates were UV sterilized after production. As part of this experimentation, methods were developed for the first time to attach PEG hydrogels to the flexible-bottom 96-well plates. The plate wells were first coated with 1 mg/ml poly-L lysine (Millipore-Sigma) + 1% penicillin/streptomycin in dPBS at 37 °C overnight. After rinsing with dPBS, the wells were then treated at 4 °C for 2 h with 2 mg/ml Thiol-PEG-NHS, 2000 Da (Nanocs) containing 1% penicillin/streptomycin in 1 mM NaH_2_PO_4_. Wells were rinsed with dPBS before a hydrogel pre-polymer solution containing suspended AVICs was placed in each well. Similar to previous reports,^11^ the pre-polymer solution contained 333 000 cells/ml, 3% 8-arm 40 kDa PEG-norbornene (Jenkem), 1 mM CRGDS adhesive peptides (Bachem), 0.05% lithium phenyl-2,4,6-trimethylbenzoylphosphinate photoinitiator (Millipore-Sigma), and 3.25 mM MMP-degradable crosslinker (Bachem) in dPBS. The pH of the pre-polymer solution was adjusted to 7 with NaOH before cells were added. The gel was cross-linked under UV light (365 nm, ∼2.5 mW/cm^2^) for 3 min. The resultant gels had stiffnesses ranging from 3.3 to 3.8 kPa (n = 3). Gels with AVICs were cultured in DMEM + 10% FBS + 1x PSG for 2 days and then cultured for another 24 h in reduced serum (1% FBS) before experimentation. Reduced serum media were changed daily during stretch conditioning on the HT-BOSS. At the end of 48 h of conditioning, samples were fixed in the 1.00 stretch configuration for 45 min with 4% paraformaldehyde in dPBS and stored at 4 °C in dPBS.

### Verification of induced PEG hydrogel and AVIC stretches

To determine if the bulk PEG gel deformed similar to that of the membrane of the specialized HT-BOSS plate, fiducial markers were dispersed evenly in wells with and without hydrogel and imaged with a Meiji dissection microscope equipped with a Nikon D3100 camera. Images were taken at varying piston height increments from 0 to 10 mm. The fiducial marker movements were tracked with Fiji/ImageJ (NIH) and analyzed to calibrate the piston height to stretch of the gel or membrane.

### AVIC nuclear shape evaluation

**FIG. 3.**
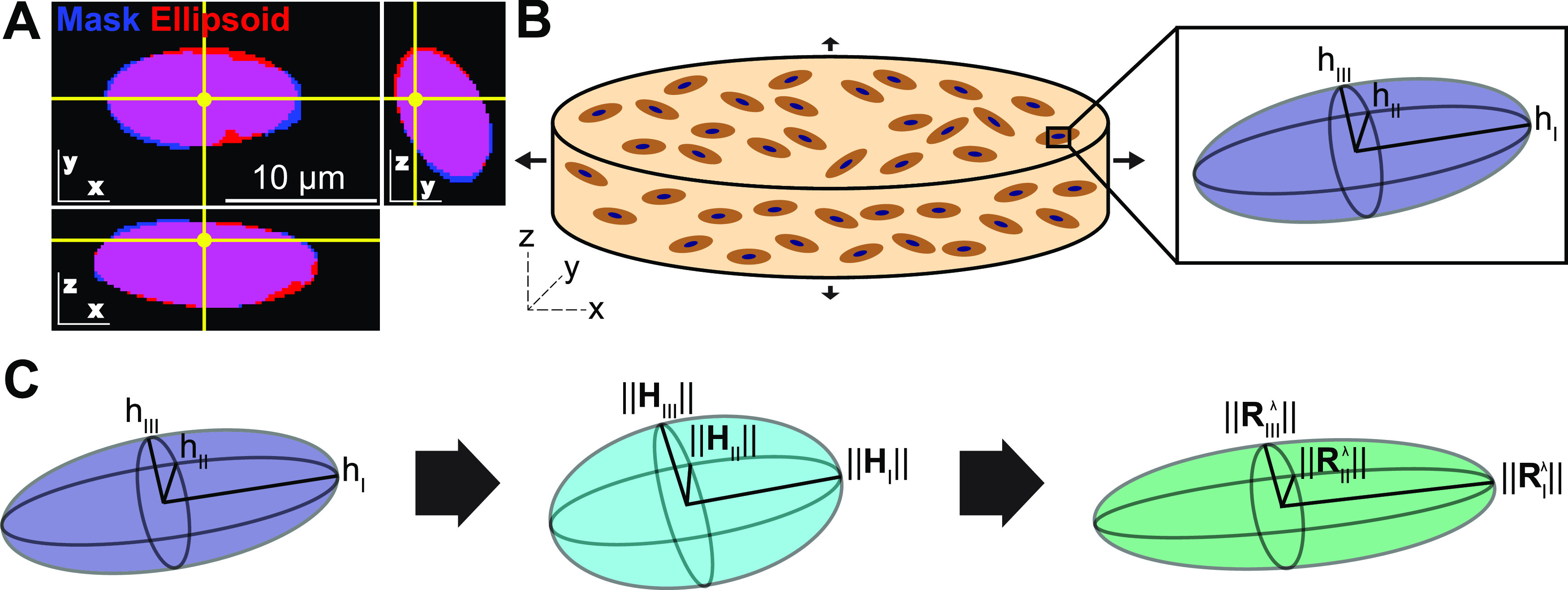
AVIC nuclear evaluation within PEG gels. (a) Overlay of the closed Otsu binarized mask of a representative AVIC nucleus (blue) and its corresponding best fit ellipsoid (red). Overlap of nuclear mask and nuclear best fit ellipsoid depicted in magenta. Yellow point in each frame is the same point in the x-, y-, and z-coordinate system. (b) Schematic of the x-, y-, and z-coordinate system implemented within the gel and the hemi-axial lengths h_I_, h_II_, and h_III_ of the best fit ellipsoid of each nucleus. The ratio of the longest to shortest hemi-axial lengths (h_I_/h_III_) and the mid-length to shortest hemi-axial lengths (h_II_/h_III_) of the best fit ellipsoid were determined to characterize the shape of the AVIC nucleus. Ratios of h_I_/h_III_ and h_II_/h_III_ at each deformation level were calculated to determine changes in 3D nuclear shape upon deformation. (c) Schematic depicting calculations made to determine the theoretical nuclear ellipsoid reference state hemi-axial lengths, $\left||H_{i}|\right|$, and the theoretical nuclear ellipsoid hemi-axial lengths at each deformation level, $\left\|R_{i}^{\lambda }\right\|$, from the measured hemi-axial values, h_i_.

To confirm that bulk deformation of the PEG hydrogels was fully transduced to the AVICs, the nuclear shape of AVICs fixed at different stretch values were evaluated. Since AVICs are highly dendritic, shape analysis of the cell membrane does not lend itself to a simple analysis. Thus, AVIC nuclear shape was utilized as a metric for overall AVIC deformation, similar to previous reports.^3,29,46^ Plates containing gels with imbedded AVICs were placed on the HT-BOSS and the pistons were set to deliver a sustained stretch while cells were fixed for 45 min with 4% paraformaldehyde in dPBS. Varying wells were exposed to varying levels of stretch between 1.025 and 1.175. Samples were removed from the plate, cut in half, and equilibrated in Hank's buffered salt solution, +Ca +Mg -phenol red (HBSS). Cell membrane staining was accomplished at 4 °C with 15 *μ*g/ml WGA-555 (Invitrogen) in HBSS and washed with HBSS. The cells were then permeabilized with 0.2% IF wash buffer (0.2% tween, 1% BSA in dPBS) overnight at 4 °C, then stained with DAPI in 0.05% IF wash buffer overnight at 4 °C and washed in 0.05% IF wash buffer. Gels were stored in dPBS at 4 °C protected from light until imaging. Since AVIC nuclei are generally ellipsoidal in shape [Fig. 3(a)],^3^ the AVIC nuclear shape was characterized as the best fit ellipsoid of the nucleus [Figs. 3(a) and 3(b)]. To determine nuclear shape from DAPI staining, coordinates denoting a bounding box for the nucleus and coordinates of background were input into the script. The nuclear mask was determined by Otsu binarization^47^ and closing within the bounding box. All image analysis was performed in the Julia programming language. As described previously,^48^ the 3D moments of the resulting nuclear mask were determined by

$$\begin{array}{c} \begin{array}{c} s_{j}^{2}=\sum \frac{{\left(j-j_{C}\right)}^{2}}{V}\;j=\left\{x,\;y,\;z\right\},\\ s_{\mathrm{xy}}=\sum \frac{\left(x-x_{C})(y-y_{C}\right)}{V}, \end{array}\\ s_{\mathrm{xz}}=\sum \frac{\left(x-x_{C})(z-z_{C}\right)}{V},\\ s_{\mathrm{yz}}=\sum \frac{\left(y-y_{C})(z-z_{C}\right)}{V}, \end{array}$$ (1)

where x, y, and z are the coordinates of each voxel of the nuclear mask, the corresponding $j_{C}$ values are the coordinates of the centroid of the mask, and V is the volume of the mask. The resulting s values were then placed into an **s** matrix

$$s=\left[\begin{array}{ccc} s_{x}^{2} & s_{\mathrm{xy}} & s_{\mathrm{xz}}\\ s_{\mathrm{xy}} & s_{y}^{2} & s_{\mathrm{yz}}\\ s_{\mathrm{xz}} & s_{\mathrm{yz}} & s_{z}^{2} \end{array}\right],$$ (2)

and the resulting eigenvalues, $\mu_{I}{,\mu }_{\mathrm{II}},\;\text{and}\;\mu_{\mathrm{III}},$ and eigenvectors, $e_{I}{,e}_{\mathrm{II}},\;\;\text{and}\;e_{\mathrm{III}}$, of **s** were determined. The hemi-axial lengths of the best fit ellipsoid, $h_{I}{,h}_{\mathrm{II}},$ and $h_{\mathrm{III}},$ were then calculated from the equation for the moment of inertia for a solid space-filled ellipsoid such that

$$h_{i}=\sqrt{{5\mu }_{i}}\;i=\{I,\mathrm{II},\mathrm{III}\}.$$ (3)

The ellipsoid hemi-axes under the imposed deformation were referred to the laboratory coordinate system x, y, and z and ordered so that $h_{I}\;{\geq h}_{\mathrm{II}}\geq h_{\mathrm{III}}$ [Fig. 3(b)].

### Verification of PEG hydrogel-induced 3D AVIC deformations

Next, predictions of nuclear shape to the measured nuclear shapes in varying stretch configurations were performed. Since the HT-BOSS system imparted an equi-biaxial stretch in the x–y plane [Figs. 2(a) and 2(b)], the in-plane components of the imposed stretch are $\lambda_{x}=\lambda_{y}=\lambda$. Next, as the PEG gel is an incompressible solid, the gel maintains a constant volume so that $\lambda_{x}\lambda_{y}\lambda_{z}=1$ and $\lambda_{z}=1/\lambda^{2}$. The resulting deformation gradient tensor, F, for this deformation is given by

$$F=\left[\begin{array}{ccc} \lambda & 0 & 0\\ 0 & \lambda & 0\\ 0 & 0 & \frac{1}{\lambda^{2}} \end{array}\right].$$ (4)

Since individual AVICs could only be fixed at one specific stretch value, the referential state (i.e., λ=1) of each AVIC could not be directly measured. However, it is possible to estimate a reference state for each AVIC by implementing **F**. This was done for each AVIC nucleus by determining

$${H_{i}=F}^{-1}h_{i}e_{i}\;i=\{I,\mathrm{II},\mathrm{III}\},$$ (5)

where | $\left|H_{i}|\right|$ are the corresponding hemi-axial lengths in the reference configuration. Ratios of ǁH_I_ǁ/ǁH_III_ǁ and ǁH_II_ǁ/ǁH_III_ǁ were then calculated to determine the referential AVIC 3D nuclear shapes. **F** was then used to estimate the nuclear shape at deformation levels, λ, between 1.025 and 1.175 at increments of 0.025 such that

$$R_{i}^{}(\lambda )=F{(\lambda )\;H}_{i}\;i=\left\{I,\mathrm{II},\mathrm{III}\right\}.$$ (6)

Ratios of $\left\|R_{I}^{\lambda }\right\|/\left\|R_{\mathrm{III}}^{\lambda }\right\|$ and $\left\|R_{\mathrm{II}}^{\lambda }\right\|/\left\|R_{\mathrm{III}}^{\lambda }\right\|$ were then calculated at each deformation level, and relations to stretch level vs the theoretically estimated nuclear ratios assuming isochoric deformations were determined.

### **α**SMA/VMN staining

To determine the level of cytoskeletal activation in AVICs conditioned for 48 h to varying stretch histories, randomly selected PEG gels were fixed in the reference state and stained for αSMA and VMN. ICC in PEG gels was employed here because polymerized cytoskeletal proteins present during AVIC activation have a very large ICC intensity signal over non-polymerized cytoskeletal monomers that are also present. Samples were removed from the HT-BOSS plate, cut in half and permeabilized with 0.05% IF wash buffer (0.05% tween-20, 1% BSA, in dPBS). Samples were then stained overnight at 4 °C in a mixture of mouse IgG2a-anti αSMA antibody (Abcam 7817) and rabbit IgG-anti VMN antibody (Cell Signaling 5741) in 0.05% IF wash buffer, washed in 0.05% IF wash buffer, stained overnight at 4 °C in a mixture of goat anti-mouse IgG2a FITC (Invitrogen 31634) and goat anti-rabbit DyLight 405 (Invitrogen 35550) in 0.05% IF wash buffer, and again washed in 0.05% IF wash buffer. Stained samples were protected from light and stored at 4 °C overnight in dPBS prior to imaging.

### SMAD 2/3, cell membrane, and nuclear staining

To determine nuclear shape and nuclear localization of SMAD 2/3 in conditioned AVICs, separate randomly selected samples that were fixed in the reference state after conditioning were removed from the plate, cut in half, and equilibrated in HBSS. Cell membranes were stained overnight at 4 °C in 15 *μ*g/ml WGA-555 (Invitrogen) in HBSS and washed at RT in HBSS. Permeabilization of the cells then occurred by incubation with 0.2% IF wash buffer (0.2% tween, 1% BSA in dPBS) overnight at 4 °C. Samples were stained with rabbit anti-SMAD2/3 (Cell Signaling 8685) in 0.05% IF wash buffer overnight at 4 °C and washed with 0.05% IF wash buffer. The samples were then stained with DAPI and goat anti-rabbit Alexafluor-488 (PIA32731) in 0.05% IF wash buffer overnight at 4 °C and washed in 0.05% IF wash buffer. Gels were stored in dPBS at 4 °C protected from light until imaging. Controls were performed by treating unconditioned samples with either dPBS or 5 ng/ml TGFβ for 45 min. before fixation in the reference configuration to confirm that changes in nuclear localization of the SMAD 2/3 signal were detectible with this staining protocol.

### Imaging

AVICs were imaged with a Nikon W1 spinning disk confocal microscope equipped with a Plan Apochromat VC 60× water lens while implementing 405, 488, and 561 excitation lasers in succession at the Center for Biomedical Research Support Microscopy and Imaging Facility at UT Austin (RRID# SCR_021756). The NIS-Elements AR 5.11.00 software powered the microscope.

### Image processing

Since this study was focusing on polymerization of cytoskeletal components, which needed to be finely resolved for proper quantification, images taken of αSMA and VMN were deconvolved with Bregman-corrected Richardson–Lucy algorithm regularized by total variation, which gives more accurate deconvolution than those of Weiner or naive Richardson–Lucy deconvolution routines.^49^ Briefly, the deconvolution task considers the following model of image formation:

$$i=P\left(o\;•\;q\right),$$ (7)

where • is the convolution operator, and image i is obtained by convolving the true object o with point spread function q, then corrupting the result with Poisson noise P. The original object can then be obtained from

o ∈argmino∈BVΩo≥0 DKLi, o • q +αoBV(Ω), (8)

where α is a positive regularization parameter, $D_{\mathrm{KL}}$ is the Kullback–Leibler functional given by

$$D_{\mathrm{KL}}=\int i\left(x\right)\cdot \log\frac{i(x)}{o\left(x\right)\;•\;q(x)}\;\mathrm{dx},$$ (9)

and ${\left|\cdot \right|}_{\mathrm{BV}(\Omega )}$ is the total variation functional defined by

$${\left|o\right|}_{\mathrm{BV}(\Omega )}=\int_{\Omega }^{\;}\left|\nabla o\right|\;\mathrm{dx},$$ (10)

with BV(Ω) denoting the space of functions bounded by total variation. This is the total variation-regularized Richardson–Lucy problem. The above problem typically suffers from contrast reduction, instead producing cartoon-like images with sharp edges. To overcome this effect, contrast enhancement via Bregman distance iterations was performed. For a convex and proper functional J, Banach space X, o ∈X, and $p\in \partial J\left(o\right)$, the generalized Bregman “distance” is defined by

$$D_{J}^{p}\left(\tilde{o},\;o\right)≔\left(\tilde{o}\right)-J\left(o\right)-\left\langle p,\;\tilde{o}-o\right\rangle ,\;\tilde{o}\in X,$$ (11)

where quotes are used since the Bregman distance violates the triangle inequality and is not generally symmetric; thus, it is not a distance in the metric sense. The original problem is then modified to incorporate contrast refinement steps in an outer loop l as

ol+1∈argmino∈BVΩo≥0 DKLi,o • q+αD⋅BVΩpl(o, ol), pl∈∂ulBV(Ω). (12)

Next, the intensity of the fluorescent signal of stained proteins acquired was obtained from the deconvolved 3D confocal images. In AVICs stained for αSMA and VMN, background intensity subtraction, Otsu binarization, and morphological closing on each channel was performed so that all portions of the signal for a given cell were a part of the channel mask. The resultant channel masks were combined and saved as the cell mask from which the cell volumes were calculated. Absolute intensity of αSMA and VMN within the cell mask were determined. In AVICs stained for the cell membrane, nucleus, and SMAD 2/3, coordinates denoting a bounding box for the nucleus and coordinates of background were input into the script. The nuclear mask was determined by Otsu binarization and closing within the bounding box. Meanwhile, the AVIC membrane channel underwent unimodal Rosin binarization because of intensity distribution differences in the membrane channel from that of the nuclear channel. The membrane channel was then morphologically closed before it was combined with the nuclear mask. Seeded region growing was then performed with initial seeds of the nuclear centroid and background coordinates. All masks were saved, and volumes calculated for auditing by the researchers. Furthermore, the SMAD 2/3 nuclear/cell intensity ratio was determined, and the nuclear 3D shape was assessed by dividing the longest nuclear hemi-axis by the shortest nuclear hemi-axis.

### Statistical analysis

All results in graphs and tables were presented as mean ± SEM. Analyses were conducted on approximately equal numbers of cells from three different hearts. N values are depicted on each graph. Brown–Forsythe and Welch ANOVAs with Dunnett T3 post-tests were performed when three groups were compared. Welch's unpaired two-tailed T-tests were conducted when two groups were compared. P < 0.05 was defined as statistically significant. Where designated, linear regression analyses were performed, and the corresponding 95% confidence intervals (CIs) or R^2^ values are listed. All statistics were calculated in GraphPad Prism 9.4.1.

## RESULTS

### Verification of PEG hydrogel-induced 3D AVIC deformations

Comparisons of the piston height verses membrane or gel stretch were used to assess how closely the PEG gels followed the underlying HT-BOSS membrane deformation. Under linear regression, the membrane slope was equal to 1.0623 (95% CI 0.8226–1.1318; n = 12 membranes), while PEG hydrogel slope was equal to 0.9772 (95% CI 0.9077–1.2169; n = 12 gels). Thus, the HT-BOSS membrane deformation was closely followed by the PEG gel layer.

In the analysis of AVIC nuclear shape with PEG gel stretch, it was first noted that all AVIC nuclei tended to be aligned to the xy-plane (longest hemi-axis was 12.01 ± 2.974° from the xy-plane). Thus, the AVICs should closely follow the in-plane PEG gel deformation. Since previous work has shown that strain distribution is largely heterogeneous in piston-driven stretch systems, and shear in the x–y plane is negligible in the body of HT-BOSS samples, we assumed isochoric deformation of the samples.^42–44^ The nuclear $h_{I}/h_{\mathrm{III}}$ and $h_{\mathrm{II}}/h_{\mathrm{III}}$ were determined in AVICs fixed at varying stretch values, and a best fit line of the measured values was determined. The nuclear $\left\|R_{I}^{\lambda }\right\|/\left\|R_{\mathrm{III}}^{\lambda }\right\|$ and $\left\|R_{\mathrm{II}}^{\lambda }\right\|/\left\|R_{\mathrm{III}}^{\lambda }\right\|$ ratios of each nucleus were then computed for varying stretch levels and fit with linear regression. No statistical differences were observed between the theoretical predictions and measured value best fit lines (Table I). These results confirm that the bulk PEG gel deformations were fully transduced to the embedded AVICs.

**TABLE I.** AVIC nuclear deformation under sustained equi-biaxial stretch.

	Slope	Intercept	R^2^		p
Stretch vs $h_{I}/h_{\mathrm{III}}$					
PEG gel model $\left\|R_{I}^{\lambda }\right\|/\left\|R_{\mathrm{III}}^{\lambda }\right\|$	3.414	−1.669	0.089 84	}	Slope p = 0.9317
Best fit to data $h_{I}/h_{\mathrm{III}}$	4.522	−2.848	0.097 15		Intercept p = 0.9866
Stretch vs $h_{\mathrm{II}}/h_{\mathrm{III}}$					
PEG gel model $\left\|R_{\mathrm{II}}^{\lambda }\right\|/\left\|R_{\mathrm{III}}^{\lambda }\right\|$	1.400	−0.6858	0.1080	}	Slope p = 0.9185
Best fit to data $h_{\mathrm{II}}/h_{\mathrm{III}}$	1.898	−0.1290	0.1177		Intercept p = 0.8969

### ***α***-Smooth muscle actin (*α*SMA) polymerization is a function of peak stretch

**FIG. 4.**
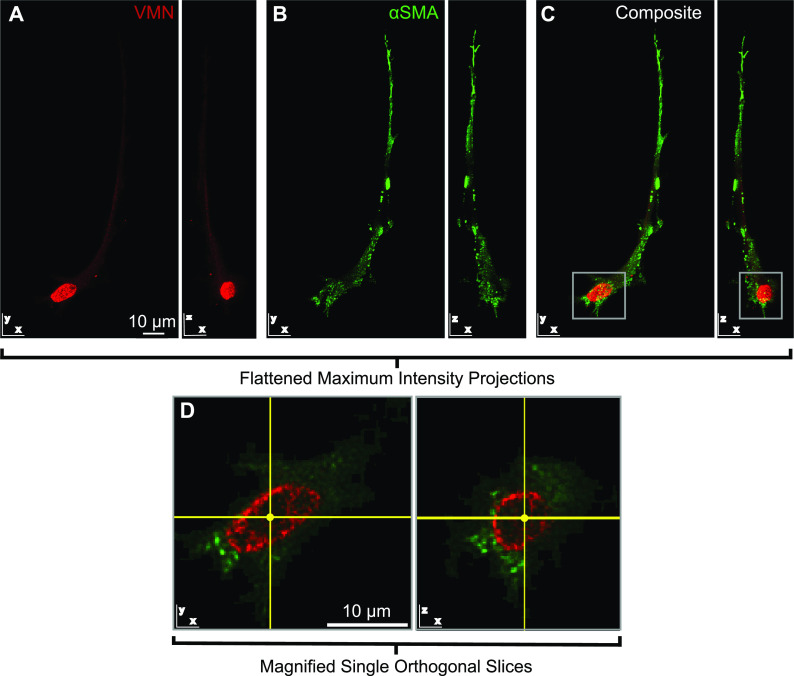
3D VMN and αSMA polymerization in cyclically stretched AVICs. (a)–(c) Maximum intensity flattened projections in the x–y plane and x–z plane for (a) VMN (red), (b) αSMA (green), and (c) the composite for a representative deconvolved image of an AVIC immunostained within a 3D PEG hydrogel. (d) Single slice and orthogonal single slice magnified image of nuclear region of the same cell; the point marked at the center of the crosshatch is the same point in 3D space in all fields of view.

**FIG. 5.**
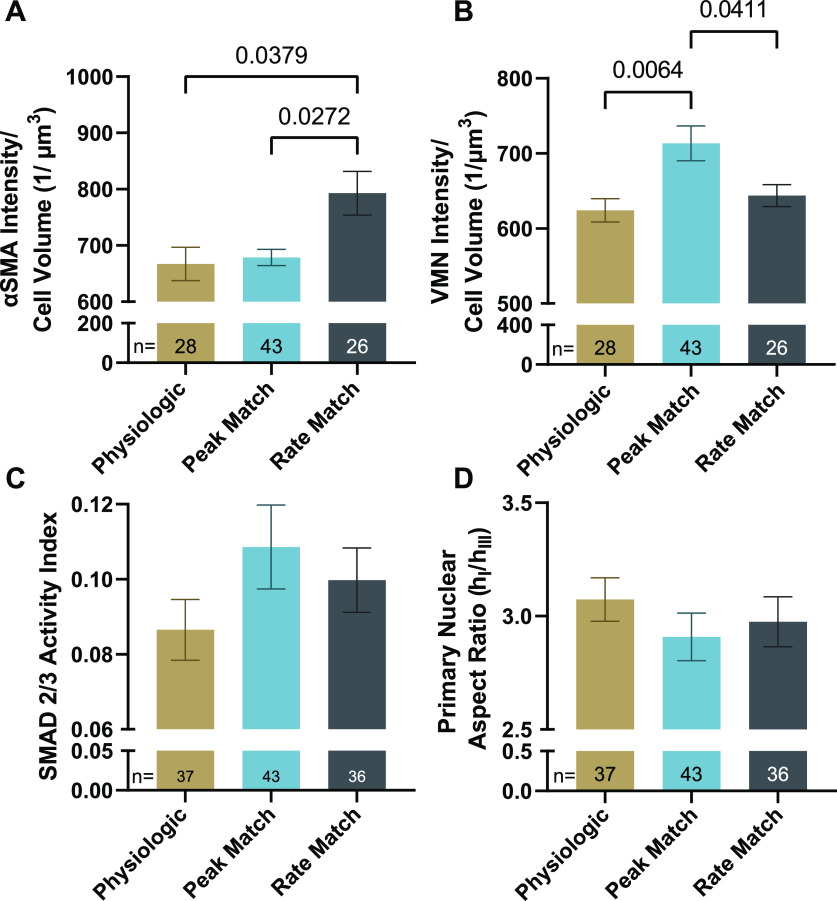
Waveform vs AVIC activation. (a) αSMA and (b) VMN intensity per cell volume in gels conditioned for 48 h with designated waveforms and fixed in the reference state. (c) SMAD 2/3 nuclear intensity divided by cell intensity from cells conditioned with indicated waveforms. (D) Primary nuclear aspect ratio measured as the longest hemi-axis divided by the shortest hemi-axis of the nuclear best fit ellipsoid from cells conditioned with indicated waveforms and fixed in the reference state.

When cells were conditioned for 48 h with varying stretch waveforms and then fixed in the reference configuration, AVICs conditioned with the rate-matching waveform had significantly higher αSMA polymerization (792.9 ± 38.65 1/*μ*m^3^) than those conditioned with the physiologic or peak matching stretch waveforms [667.2 ± 29.54 and 678.6 ± 14.40 1/*μ*m^3^, respectively; Figs. 4 and 5(a)]. Since the rate-matching waveforms had a lower peak stretch of 1.062 than the physiologic and peak matching waveforms of 1.1, the data suggest that a key parameter of αSMA polymerization in AVICs is peak stretch level, where variation from physiological levels to those seen during disease favor higher polymerization.

### Vimentin (VMN) polymerization

AVICs conditioned with the peak matching waveform expressed statistically higher VMN polymerization (713.4 ± 23.26 1/*μ*m^3^) than AVICs exposed to the physiologic or rate-matching waves [624.1 ± 15.52 and 643.8 ± 14.75 1/*μ*m^3^, respectively; Figs. 4 and 5(b)]. This suggests that stretch rate is an important factor in VMN polymerization since AVICs conditioned in a slower than physiological stretch rate develop more VMN fibers.

### SMAD 2/3 activity

**FIG. 6.**
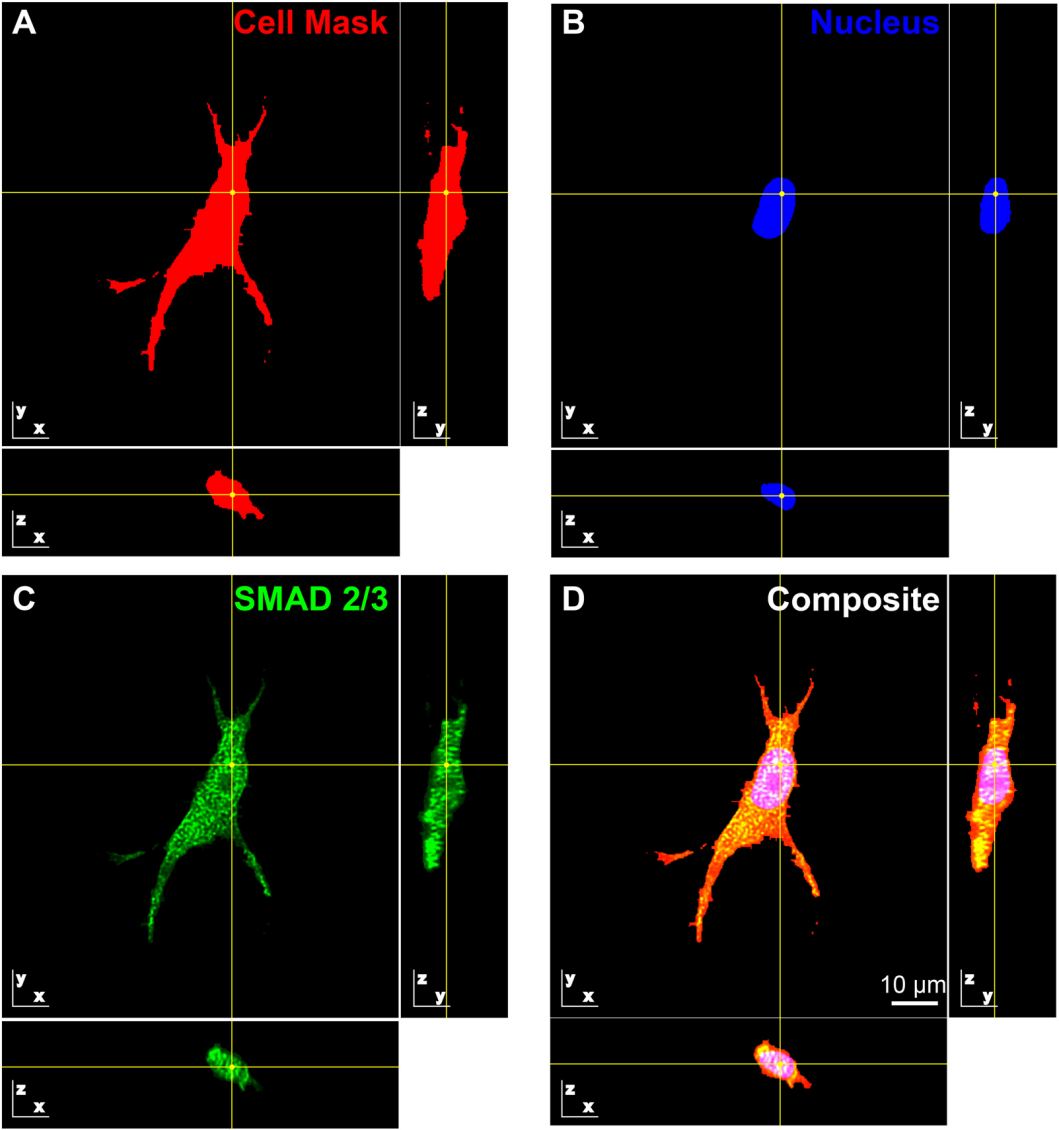
3D SMAD 2/3 localization. (a)–(d) Representative deconvolved image of an AVIC that was immunostained within the fixed 3D PEG hydrogel. Orthogonal single slices in the x–y, x–z, and y–z planes are depicted for (a) cell membrane (red), (b) nucleus (blue), (c) SMAD 2/3 (green), and (d) the composite; the point marked at the center of the crosshatch is the same point in 3D space in all fields of view. Nuclear localization of SMAD 2/3 is indicative of its activation.

A general trend was found that SMAD 2/3 nuclear localization increased in AVICs conditioned with the peak matching wave (0.1086 ± 0.011 20) more so than AVICs made manifest from the physiologic (0.086 51 ± 0.008 121) and rate matching (0.099 74 ± 0.008 577) waveforms [Figs. 5(c) and 6].

### Nuclear shape in the reference state after conditioning

While both the post-conditioned nuclear volume and cell volume were unaffected by the waveform type (Table II), 3D nuclear shape exhibited a trend of being dependent on stretch rate, with the peak matching waveform having AVICs with a more eccentric shape (2.908 ± 0.1045) than those conditioned with physiologic (3.073 ± 0.095 63) or rate matching (2.975 ± 0.1103) waveforms [Fig 5(d)]. This suggests that a reduced non-physiological stretch rate like that seen in BAV patients may correlate with a decreased 3D nuclear elongation.

**TABLE II.** Reference state cell and nuclear volume after cyclic stretch conditioning. No significant differences between any groups.

Waveform type	Cell volume			Nuclear volume	
Physiologic	7116 ± 836.6 *μ*m		n = 37	510.5 ± 32.87 *μ*m	n = 37
Peak matching	6863 ± 478.4 *μ*m		n = 43	493.2 ± 30.86 *μ*m	n = 43
Rate matching	6724 ± 539.5 *μ*m		n = 36	550.0 ± 31.84 *μ*m	n = 36

### Cyclical stretch increases AVIC volume and decreases **α**SMA polymerization

Interestingly, stretch conditioning, in general, increased the reference state nuclear and cell volumes when compared to AVICs grown in static culture. This was true even though SMAD 2/3 activity was not markedly different in AVICs from static conditions. In contrast, αSMA polymerization is significantly higher in static 3D cultures than the average cyclically conditioned AVIC (Table III). This suggests that AVIC size is regulated by cyclic stretch in a SMAD-independent manner and that AVICs held under static 3D conditions are more likely to have certain aspects of an activated phenotype.

**TABLE III.** 3D static vs 3D cyclically conditioned reference state averages.

Quantity	Static culture		Cyclically stretched		p
AVIC volume	3944 ± 354.2 *μ*m	n = 40	7303 ± 430.3 *μ*m	n = 116	<0.0001
Nuclear volume	413.1 ± 18.98 *μ*m	n = 40	489.0 ± 19.02 *μ*m	n = 116	0.0055
αSMA polymerization	873.3 ± 25.13 1/*μ*m^3^	n = 31	705.9 ± 15.63 1/*μ*m^3^	n = 97	<0.0001
VMN polymerization	682.0 ± 18.05 1/*μ*m^3^	n = 31	669.0 ± 12.51 1/*μ*m^3^	n = 97	0.5551
SMAD 2/3 activity	0.099 30 ± 0.008 963	n = 40	0.098 80 ± 0.005 589	n = 116	0.9626
Nuclear shape (*h_I_/h_III_*)	2.734 ± 0.1276	n = 40	2.982 ± 0.059 85	n = 116	0.0845

## DISCUSSION

### Study rationale

Native valve leaflets have their ECM arranged so that collagen fibers align primarily in the circumferential direction.^35,50^ Importantly, the collagen fibers play an important role in the deformation of the AVICs.^3,29,46,51^ Since stretch of the collagen in the circumferential direction is the primary tissue level deformation metric of AVIC deformation in the native valve and major decreases in circumferential stretch occur in BAV leaflets,^1,2,5^ a focus was placed in this study on understanding how the properties of cyclic circumferential stretch affected AVIC activation. In addition, all heart valves undergo large deformations and experience very high stretch rate during the opening and closing phases. Such tissue level deformations in valves are transduced to the underlying VIC populations.^3,29,46,52^ Yet, the effects these unique large deformation and deformation rate patterns have on VIC function was unknown. The present study was, thus, a first attempt to address this knowledge gap.

The PEG gel system employed herein is an accepted simulacrum for ECM as it mimics many of the biochemical and mechanical cues of the native tissue.^12,38^ Implementation of PEG gels here allowed for a thorough analysis of stretch of cells in 3D while being able to hold constant confounding factors such as stiffness and number of integrin binding sites. Although the system employed in the present study did not have a fibrous ECM structure that undergoes anisotropic deformations as in aortic valve leaflets, the study was designed to allow for systematic studies not possible with native tissues. The cyclic stretch waveforms employed were designed to allow for a separation of the influences of stretch rate, peak stretch, and RMS stretch history on AVIC activation in a 3D setting. This study, for the first time, was therefore able to demonstrate the effects of differences in stretch history on AVIC activation.

### Role of VMN in mechanical shielding of the AVIC nucleus

**FIG. 7.**
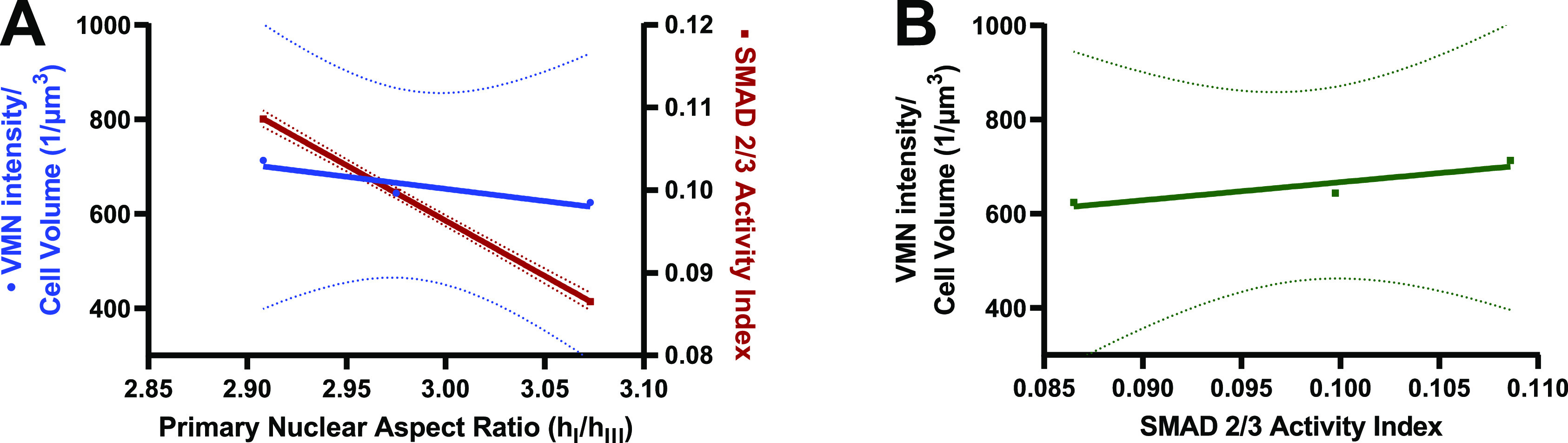
Strong correlations between VMN polymerization, SMAD 2/3 activity, and nuclear shape. (a) Average nuclear eccentricity correlated with average VMN intensity per cell volume (y = −516.2× + 2201, and R^2^ = 0.8336) and average SMAD 2/3 activity (y = −0.1340x+0.4982, and R^2^ = 1.000) from AVICs conditioned for 48 h of stretch waveforms. (b) Average SMAD 2/3 nuclear localization correlated with average VMN intensity per cell volume from AVICs conditioned for 48 h of stretch waveforms (y = 3843x + 282.7, and R^2^ = 0.8294). Dotted lines represent 95% confidence intervals. These trends suggest that a feedback mechanism may exist whereby mechanical stretch inputs affect a system where SMAD 2/3 activity is held in check by VMN polymerization and nuclear shape.

Due to the similar trends found between VMN polymerization, SMAD 2/3 activity, and nuclear shape, paired with prior publications outlining fibroblasts in 3D^21^ and current imagery here of AVICs depicting caging of the nucleus by polymerized VMN [Fig. 4(d)], it was determined if there was a correlation between each of these parameters. Indeed, as can be seen in Fig. 7, there is a correlation between the average values of VMN, SMAD 2/3, and nuclear shape. Future study to determine if a feedback mechanism may exist whereby mechanical stretch inputs affect a system where SMAD 2/3 activity increases, causing greater VMN polymerization, which leads to a more eccentric nucleus that aids in blocking further SMAD 2/3 activity is thereby warranted. This could partially be the mechanism by which AVICs maintain homeostasis in a more quiescent state when small changes in the mechanical environment occur. It has recently been reported that calcific phenotype AVICs from CAVD patients have aberrant nuclear morphologies, with shapes of these nuclei looking like cups or beads on a string.^32^ In the current work starting with healthy porcine AVICs, there was no evidence of oddly shaped nuclei, possibly because the stretches and conditions they were subjected to did not promote a calcific phenotype. Nevertheless, changes in elongation here were measurable and trended toward more nuclear eccentricity in the reference state being associated with non-physiological rates of stretch conditioning.

### Complex interactions within AVIC activation

It was found here that two important aspects of AVIC activation, αSMA polymerization and VMN polymerization, are not directly tied to one another. Caution should, therefore, be taken when reducing VIC activation to an evaluation of αSMA alone. In the present study, SMAD 2/3 activity correlated well with VMN signal but not with αSMA levels. Although αSMA and VMN transcription can both be activated by SMAD 2/3, other signaling systems are integrated within the nucleus to have SMAD complexes only activate certain genes under certain conditions.^53,54^ In addition, polymerization of αSMA and VMN are regulated within the cytoplasm by two completely different systems. Therefore, future study into the integration of these signals is well warranted.

In the present study, cyclically stretching AVICs with any stretch waveform led to an increase in nuclear and cell volume and a decrease in αSMA polymerization when compared to static cultures. Similar changes in αSMA between static and cyclically stretched pig AVICs in collagen gels were also seen in Ref. 24, but no differences were seen among stretch conditions in dog MVICs in collagen gels in Ref. 25. Other variables important to VIC activation, such as stiffness of the gels^55,56^ and number of integrin binding sites,^57^ are also different between these studies. In the current work, stiffness and CRGDS concentrations were held constant in the gels so that attention could be focused on stretch properties. The conditions held constant allowed for changes in activation to be seen between static and differentially stretched samples. It is possible that at different stiffnesses or with differing numbers of integrin binding sites, the effects of cyclic stretch conditioning could be swamped by inputs from these other variables. Future investigation of how changes in stiffness and fiber alignment affect activation in a given AVIC may help shed light on location-specific alterations that occur in valve disease, since the ventricularis layer has a circumferential stiffness of 7.41 kPa and a radial stiffness of 3.68 kPa, and the fibrosa layer has a circumferential stiffness of 13.02 kPa and a radial stiffness of 4.65 kPa.^58^ Furthermore, understanding of the integration of these multiple mechanical inputs through modeling is, therefore, an exciting avenue of research for the future.

### Limitations

The changes in expression of previously associated proteins with VIC to myofibroblast transition include increases in collagens, elastins, prostaglandins, MMPs, and TIMPs.^8^ All of these proteins are important in the production of or remodeling the ECM of the valve tissue and are upregulated during stenosis and CAVD. That they are upregulated in activated AVICs again depicts the importance of understanding the biomechanical–biochemical interaction on a cellular level. Because these substances are excreted by AVICs, attempts to stain these effectively in the gels were not quantitative due to the nature of ICC protocols requiring multiple wash steps. Correlating VIC internal activation state with its secretory function has previously been completed on an mRNA transcript level,^32,36^ but future investigation into how stretch in 3D environments affects ECM secretion is still on the horizon.

### Summary and conclusions

Through the development and validation of a method to implement equi-biaxial deformation in the HT-BOSS with 3D cultures, an evaluation of mechanical characteristics of stretch history revealed that differing aspects of stretch were essential for distinct AVIC activation behaviors. Peak stretch had a significant effect on αSMA polymerization, while stretch rate was the significant effector of VMN polymerization. Lower than physiological levels of peak stretch or stretch rate, which are seen in BAVs and diseased TAVs, are significantly predictive of AVIC activation behaviors. Furthermore, evidence suggests that VMN may be controlled by a negative feedback loop between SMAD 2/3 activity, VMN expression, and nuclear shape, while no such evidence could be seen for SMAD 2/3 activity playing a role in αSMA polymerization under the tested circumstances. This highlights the need for future study, which focuses on a systems integration level, so that interrelationships between multiple mechanical and biochemical components can be addressed. Future studies will, therefore, focus on integrating information learned about the impact of waveform properties on the differences seen between stretches observed in TAV and BAV patients to determine how patient-derived waveforms affect various transcription factor activity and cytoskeletal polymerization within patient-derived AVICs.

## Data Availability

The data that support the findings of this study are available from the corresponding author upon reasonable request.
